# Additional oxidative stress reroutes the global response of *Aspergillus fumigatus* to iron depletion

**DOI:** 10.1186/s12864-018-4730-x

**Published:** 2018-05-10

**Authors:** Vivien Kurucz, Thomas Krüger, Károly Antal, Anna-Maria Dietl, Hubertus Haas, István Pócsi, Olaf Kniemeyer, Tamás Emri

**Affiliations:** 1 Department of Biotechnology and Microbiology, Faculty of Sciences and Technology, University of Debrecen, Egyetem tér 1, Debrecen, H-4032 Hungary; 2 Molecular and Applied Microbiology, Leibniz Institute for Natural Product Research and Infection Biology – Hans Knöll Institute (HKI), 07745 Jena, Germany; 3 Department of Zoology, Faculty of Sciences, Eszterházy Károly University, Eszterházy tér 1, Eger, H-3300 Hungary; 4 Division of Molecular Biology, Biocenter, Medical University of Innsbruck, A6020 Innsbruck, Austria

**Keywords:** *Aspergillus fumigatus*, Combinatorial stress, Iron deprivation, Oxidative stress, Proteomics, Stress response, Transcriptomics

## Abstract

**Background:**

*Aspergillus fumigatus* has to cope with a combination of several stress types while colonizing the human body. A functional interplay between these different stress responses can increase the chances of survival for this opportunistic human pathogen during the invasion of its host. In this study, we shed light on how the H_2_O_2_-induced oxidative stress response depends on the iron available to this filamentous fungus, using transcriptomic analysis, proteomic profiles, and growth assays.

**Results:**

The applied H_2_O_2_ treatment, which induced only a negligible stress response in iron-replete cultures, deleteriously affected the fungus under iron deprivation. The majority of stress-induced changes in gene and protein expression was not predictable from data coming from individual stress exposure and was only characteristic for the combination of oxidative stress plus iron deprivation. Our experimental data suggest that the physiological effects of combined stresses and the survival of the fungus highly depend on fragile balances between economization of iron and production of essential iron-containing proteins. One observed strategy was the overproduction of iron-independent antioxidant proteins to combat oxidative stress during iron deprivation, e.g. the upregulation of superoxide dismutase Sod1, the thioredoxin reductase Trr1, and the thioredoxin orthologue Afu5g11320. On the other hand, oxidative stress induction overruled iron deprivation-mediated repression of several genes. In agreement with the gene expression data, growth studies underlined that in *A. fumigatus* iron deprivation aggravates oxidative stress susceptibility.

**Conclusions:**

Our data demonstrate that studying stress responses under separate single stress conditions is not sufficient to understand how *A. fumigatus* adapts in a complex and hostile habitat like the human body. The combinatorial stress of iron depletion and hydrogen peroxide caused clear non-additive effects upon the stress response of *A. fumigatus*. Our data further supported the view that the ability of *A. fumigatus* to cause diseases in humans strongly depends on its fitness attributes and less on specific virulence factors. In summary, *A. fumigatus* is able to mount and coordinate complex and efficient responses to combined stresses like iron deprivation plus H_2_O_2_-induced oxidative stress, which are exploited by immune cells to kill fungal pathogens.

**Electronic supplementary material:**

The online version of this article (10.1186/s12864-018-4730-x) contains supplementary material, which is available to authorized users.

## Background

*Aspergillus fumigatus* is a ubiquitous fungal species, which occurs commonly on decaying organic matter and in soil under a wide variety of conditions [1, 2]. This mould is also known as one of the most important airborne human pathogenic fungi with an outstandingly high mortality rate (50–95%) in immunocompromised patients, who suffer from an invasive *A. fumigatus* infection (referred to as invasive aspergillosis) [3–6]. The reasons for the unique success of *A. fumigatus* as the most important opportunistic human pathogen among phylogenetically closely related aspergilli are largely unknown.

Several fungal factors that determine the outcome of infections have been identified [6–10]. These include the rodlet and cell wall melanin layers of conidia [11, 12]; the cell wall exopolysaccharide galactosaminogalactan, which has possible anti-inflammatory effects [13]; the production of mycotoxins such as the immune response modulator gliotoxin [14]; elastinolytic proteases [15]; efficient iron and zinc acquisition systems [16, 17]; acquisition and detoxification of copper [18, 19]; as well as the suitable oxidative stress defense systems to detoxify reactive oxygen species (ROS) generated by macrophages and neutrophils [10, 20].

The stress responses of *A. fumigatus* have also been studied intensively in order to identify a potential Achilles’ heel of this pathogen. Researchers aimed to elucidate the orchestration of the signaling network regulating the stress response and understanding the physiological background of the adaptation process [21–29]. Iron starvation and oxidative stress are typical stresses for *A. fumigatus* and also for other microorganisms, which they may encounter in the human host [29]. *A. fumigatus* acquires iron by low affinity iron transporters, the reductive iron assimilation (RIA) system, and siderophore-mediated iron uptake [30]. It is unable to utilize iron directly from human iron-binding proteins like hemoglobin, transferrin, or ferritin [30], however siderophores can chelate iron from host proteins [31]. The significance of the low affinity iron transport has not been studied in details so far [29, 30]. Both RIA and siderophore-mediated iron uptake are important in adaptation to iron starvation, however, only the contribution of siderophore biosynthesis and ferri-siderophore transport to virulence has been demonstrated until now [30, 32–34]. The transcription factor HapX is crucial in regulating the iron starvation stress response in *A. fumigatus* [35]. It down-regulates iron-consuming pathways such as iron-sulfur cluster assembly, heme biosynthesis, respiration, the tricarboxylic-acid (TCA) cycle and amino acid metabolism, while it up-regulates iron acquisition via siderophore biosynthesis [35].

Several elements of the oxidative stress response have been identified in *A. fumigatus,* which include the production of catalases, superoxide dismutases (SODs), elements of the thioredoxin and glutathione-glutaredoxin system, as well as the conidial pigment melanin [12, 26]. Both MpkA- and SakA-mediated MAPK stress signaling pathways as well as the Yap1 and Skn7 transcriptional regulators were demonstrated to modulate the oxidative stress response [36–39]. Single gene deletions had no impact on virulence, suggesting that a significant level of redundancy exists in the oxidative stress defense system of *A. fumigatus* [29]. Importantly, oxidative stress response and iron metabolism are tightly linked; iron overload can catalyze the formation of ROS, but detoxification of ROS by heme peroxidases and catalases requires the iron-containing cofactor heme [40, 41].

In natural habitats like the human body, microbial pathogens have to cope with combinations of various and rapidly changing stress conditions rather than single stress types. The interplay between different stress responses can decrease synergistically and markedly the fitness and, hence, the chance of the microorganisms to survive in the host. Indeed, human polymorphonuclear leukocytes have been shown to inhibit fungal growth by oxidative and non-oxidative mechanisms including iron depletion mediated via lactoferrin [42, 43].

Here, we examined how *A. fumigatus* adapts to combined stresses (iron deprivation/oxidative stress: -Fe/+H_2_O_2_) at the level of transcriptome and proteome. For this purpose, we compared the early stress responses to H_2_O_2_-induced oxidative stress in both iron-deprived and iron-replete *A. fumigatus* cultures. Oxidative and iron deficiency stress were chosen for two reasons: i) fungal pathogen species have to face both of them concomitantly in the human body [16, 26], and ii) combating these combined stresses is likely a difficult situation for the fungus to handle because iron is an important cofactor for several antioxidant enzymes such as catalases and heme peroxidases [40, 41] and, hence, a synergism between iron deprivation and oxidative stresses is predictable. To get comprehensive insights into the stress responses of iron-deprived *A. fumigatus* cultures towards ROS, we used a combined transcriptomic-proteomic based approach. Another reason for this is the fact that transcriptional changes do not necessarily correspond to changes in the protein concentrations [44]. This may be particularly true to iron-deprived conditions: up-regulation of an iron-dependent enzyme at the transcriptional level may not necessarily result in an increased abundance of the corresponding protein due to the lack of iron cofactors.

## Methods

### Strain and growth conditions

*A. fumigatus* strain Af293 (CBS 101355), received from the CBS-KNAW culture collection (http://www.westerdijkinstitute.nl/Collections/), was used throughout this study with exception of the oxidative stress resistance analysis and was maintained on Barrat’s minimal nitrate agar plates [45]. Plates were incubated at 37 °C for 5 d. Conidia obtained from the 5-days-old cultures were used in these experiments. Modified Barrat’s minimal nitrate broths (100 ml in 500 ml flasks) were inoculated with freshly isolated conidia and were incubated at 37 °C and 3.7 Hz shaking frequency. The modified Barrat’s minimal nitrate broth differed from the standard Barrat’s minimal nitrate medium in the added trace element solution, which did not contain EDTA and iron. This modified form of Barrat’s minimal nitrate broth was either supplemented with 30 μM FeCl_3_ (+Fe; iron-replete cultures) or used without addition of any iron (-Fe; iron-depleted cultures). Cultures (+Fe and -Fe) were inoculated with 4 × 10^8^ and 8 × 10^8^ conidia and were incubated for 33 h and 50 h, respectively, before harvest. At the two time points, the +Fe and -Fe cultures showed similar residual glucose content as well as similar dry cell mass. Oxidative stress was induced by addition of H_2_O_2_ (at 3 mM final concentration) to the cultures 1 h before sampling (+H_2_O_2_ and -H_2_O_2_ cultures).

For analysis of oxidative stress resistance, *A. fumigatus* strain ATCC 46645 (wild-type strain with similar oxidative stress resistance to the AF293 strain) and its siderophore-deficient derivative (*ΔsidA*) [30] were used. Strains (10^4^ conidia) were point-inoculated on minimal agar plates with different iron supply (30 μM FeSO_4_ or without addition of iron) with and without agents for inducing oxidative stress (1–3 mM H_2_O_2_, 0.5–2 mM paraquat or 0.01–0.03 mM menadione). Notably, ferrous iron is quickly oxidized to ferric iron during aerobic conditions, and we did not observe differences in growth patterns between supplementation with ferrous (FeSO_4_) compared to ferric (FeCl_3_) iron (data not shown). Certain plates also contained 0.2 mM bathophenanthroline disulfonate (BPS), a ferrous iron-specific chelator, which inactivates reductive iron assimilation [30]. All experiments were carried out in triplicates and growth was scored after incubation for 48 h at 37 °C.

### 2′,7′-dichlorofluorescin diacetate and chrome azurol S (CAS) liquid assays

In order to test how stress treatments perturb the redox homeostasis and siderophore production of cells, the following assays were applied: formation of 2′,7′-dichlorofluorescein (DCF) from 2′,7′-dichlorofluorescin diacetate, a marker of redox imbalance, was recorded as described previously [46]. The siderophore content of the fermentation broth was measured by the CAS liquid assay according to Machuca and Milagres [47].

### Reverse-transcription quantitative real-time polymerase chain reaction (RT-qPCR) assays

Total RNA was isolated from lyophilized mycelia according to Chomczynski [48]. RT-qPCR experiments were carried out as described previously [49] with the primer pairs presented in Additional file 1. RT-qPCR assays were carried out at the Genomic Medicine and Bioinformatics Core Facility, Department of Biochemistry and Molecular Biology, Faculty of Medicine, University of Debrecen, Debrecen, Hungary. The ΔΔCP values were used to quantify relative transcription levels and the gene Afu6g12400 (*fks1*) was selected as reference.

### RNA sequencing

Total RNA was isolated from +Fe/-H_2_O_2_ (iron-replete), +Fe/+H_2_O_2_ (H_2_O_2_-treated),-Fe/-H_2_O_2_ (iron-deprived), and -Fe/+H_2_O_2_ (iron-deprived-H_2_O_2_-treated) cultures in three biological replicates as described previously [48]. RNA samples were isolated for RNA sequencing and for RT-qPCR from independent experiments. RNA-sequencing (from library preparation to generation of fastq.gz files) was carried out at the Genomic Medicine and Bioinformatic Core Facility, Department of Biochemistry and Molecular Biology, Faculty of Medicine, University of Debrecen, Debrecen, Hungary. cDNA libraries for RNA-Seq were generated from 1 μg total RNA using a TruSeq RNA Sample Preparation Kit (Illumina, San Diego, CA, USA) according to the manufacturer’s protocol. Fragment size distribution and molarity of libraries were checked on Agilent BioAnalyzer DNA1000 chip (Agilent Technologies, Santa Clara, CA, USA). A single-read 50 bp sequencing run was performed on an Illumina HiScan SQ instrument (Illumina, San Diego, CA, USA). Each library pool was sequenced in one lane of a sequencing flow cell, and 16–18 million reads per sample were obtained. The CASAVA software was used for pass filtering and demultiplexing processing.

Reads were aligned to *A fumigatus* (Af293) genome (Genome: A_fumigatus_Af293_version_s03-m05-r04_chromosomes.fasta.gz;  http://www.aspergillusgenome.org/download/sequence/A_fumigatus_Af293/archive; Genome features file (GFF): A_fumigatus_Af293_version_s03-m05-r04_features_with_chromosome_sequences.gff.gz; http://www.aspergillusgenome.org/download/gff/A_fumigatus_Af293/archive) using tophat (version 2.0.9) [50]. Calculation of FPKM (fragments per kilobase per million mapped fragments) values at the gene level and differential gene expression testing was performed using cuffdiff (version 2.2.1) [51] based on the mappings and corresponding genome features.

### Evaluation of transcriptome data

Up-regulated, down-regulated, and stress responsive genes were defined as genes that showed significantly different expression in the three biological replicates employing the cuffdiff software (version 2.2.1) [51] (adjusted *p*-value < 0.05) and where the transcriptional difference was at least two-fold: log_2_FC > 1 (up-regulated genes), log_2_FC < − 1 (down-regulated genes) or |log_2_FC| > 1 (stress responsive genes), where FC (“fold-change”) stands for I_treated_/I_reference_ and I is the mean FPKM value).

### Proteomics sample preparation

Three biological replicates of *A. fumigatus* Af293 mycelium samples from four different growth conditions were measured as described above in the section “Strain and growth conditions”. These included iron-replete conditions with (+Fe/+H_2_O_2_) and without (+Fe/-H_2_O_2_) oxidative stress induced by 3 mM hydrogen peroxide, as well as iron-deprived conditions with (-Fe/+H_2_O_2_) and without (-Fe/-H_2_O_2_) oxidative stress. Proteins were isolated and digested as described previously [52]. Labeling of tryptic peptides with iTRAQ 4-plex (Sciex, Darmstadt, Germany) reagents was performed according to the manufacturer’s manual. Each biological replicate was represented in one 4-plex reaction. The three 4-plex reactions were made up as follows: 114 (+Fe #1; -Fe/+H_2_O_2_ #2; +Fe/+H_2_O_2_ #3), 115 (-Fe #1; +Fe #2; -Fe/+H_2_O_2_ #3), 116 (+Fe/+H_2_O_2_ #1; -Fe #2; +Fe #3), 117 (-Fe/+H_2_O_2_ #1; +Fe/+H_2_O_2_ #2; -Fe #3). Each 4-plex reaction was combined, dried (speed vac), and resolubilized in 40 μL of 0.05% (*v*/v) trifluoroacetic acid in 2/98 (v/v) acetonitrile (ACN)/H_2_O for LC-MS/MS analysis.

### Liquid chromatography coupled to tandem mass spectrometry (LC-MS/MS) analysis

Proteome analysis was performed on an Ultimate 3000 nano RSLC/QExactive Plus LC-MS/MS system (Thermo Fisher Scientific). Each 4-plex reaction was measured in triplicate with an injection volume of 4 μL. Peptides were measured as described previously [52] including the following changes: The mobile phase consisted of eluent A [0.1% (v/v) formic acid in H_2_O] and eluent B [0.1% (v/v) formic acid in a ratio of 90/10 ACN/H_2_O]. Gradient elution was 0–4 min at 4% B, 150 min at 14% B, 200 min at 19% B, 300 min at 42% B, 319–329 min at 90% B, and 330–400 min at 4% B. Higher-energy collisional dissociation (HCD) fragmentation with the help of nitrogen gas occurred at a normalized collision energy of 34 V. Dynamic exclusion of precursor ions was set to 35 s and the fixed first mass was set to m/z 110 to match the iTRAQ reporter ions (m/z 114–117).

### Protein database search and reporter ion quantification

Thermo raw files were processed by Proteome Discoverer 1.4 (Thermo). MS/MS spectra were searched against the AspGD protein database of *A. fumigatus* Af293 (www.aspergillusgenome.org/download/sequence/A_fumigatus_Af293/current/A_fumigatus_Af293_current_orf_trans_all.fasta.gz; [53]) using Mascot 2.4 (Matrix Science, UK), Sequest HT and MS Amanda including up to two missed tryptic cleavages, a precursor mass tolerance of 10 ppm, and a fragment mass tolerance of 0.02 Da. Dynamic modifications were oxidation of Met and iTRAQ labeling of Tyr residues (not considered for quantification). Static modifications were carbamidomethylation of Cys by iodoacetamide and iTRAQ labeling of Lys residues and the peptide N-terminus. At least 2 peptides per protein and a strict target false discovery rate of < 1% were required for positive protein hits. Reporter ion quantification was based on an integration tolerance of 10 ppm using the most confident centroid. Reporter ion ratios were calculated for each 4-plex reaction based on the following comparisons: –Fe/+Fe, +Fe + H_2_O_2_/+Fe, -Fe + H_2_O_2_/+Fe, -Fe + H_2_O_2_/+Fe + H_2_O_2_, -Fe + H_2_O_2_/-Fe. Only unique peptides were considered for quantification. Isotopic correction and protein median normalization was applied. The significance threshold for iTRAQ ratios were ≥ 1.5 (up- or down). The data was further manually evaluated based on the average reporter ion count (≥2 for medium confidence, ≥4 for high confidence). Furthermore, the average variability was observed as a function of the differential regulation and the precursor ion count.

### Functional annotation of transcriptome and proteome data

The FungiFun2 package (https://elbe.hki-jena.de/fungifun/fungifun.php), with default settings was used to test the enrichment of genes related to FunCat and KEGG pathway categories in stress responsive gene groups [53]. Enrichment analysis was also carried out with the AspGD Gene Ontology Term Finder (http://www.aspergillusgenome.org/cgi-bin/GO/goTermFinder; [54]) applying default settings and biological process ontology GO terms. Only hits with *p*-value < 0.05 were taken into consideration during the evaluation process. Protein enrichment analysis was carried out as described above using the appropriate gene IDs instead of protein IDs.

Fisher’s exact test (*p* < 0.05) was used to detect significant gene enrichment of the following gene/protein groups in the stress responsive gene sets:

1. Antioxidant enzymes. This group of proteins/genes contains known and putative/probable catalases, peroxidases, SODs, peroxiredoxins, glutaredoxins, thioredoxins etc. collected from *Aspergillus* Genome Database (http://aspergillusgenome.org/).
2. TCA cycle. This group contains all the proteins/genes related to the mentioned biochemical pathways according to Flipphi et al. [55].
3. Iron transport. This gene group was created according to Haas [16] and to the *Aspergillus* Genome Database using the related GO terms and their child terms.
4. Squalene - ergosterol pathway. The gene group was generated according to Alcazar-Fuoli and Mellado [56]. This pathway does not contain the first steps of sterol biosynthesis that are shared with other pathways, e.g. with siderophore biosynthesis.
5. Heme binding. This group contains the proteins/genes belonging to the “Heme binding” FunCat term according to FungiFun2 webpage [53].
6. Respiration,
7. Cu^2+^ transport,
8. Zn^2+^ transport,
9. Fe-S cluster assembly,
10. Heme biosynthesis”,
11. Drug transmembrane transport,
12. Iron-sulfur cluster binding and
13. Transcription factors. These groups of proteins/genes were constructed based on the *Aspergillus* Genome Database using the related GO terms and their child terms. Genes/proteins presented in the “TCA cycle” group were omitted from the “Respiration” group.
14. Secondary metabolite cluster genes/proteins were collected according to Inglis et al. [57] and Lin et al. [58]. Only genes of clusters determined either manually or experimentally were involved in the analysis. Key genes were defined as secondary metabolite cluster genes encoding transcription factors (TFs), non-ribosomal peptide synthases (NRPSs), polyketide synthases (PKSs), or dimethylallyl tryptophan synthases (DATS). A secondary metabolite biosynthetic gene cluster was considered as up- or down-regulated if the up- or down-regulated genes within the cluster were significantly (*p* < 0.05) enriched in the studied gene set according to the Fisher’s exact test.

## Results

### Comparison of transcriptomic and proteomic data

**Fig. 1:**
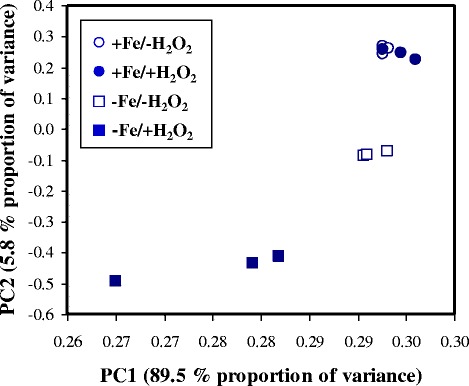
Principal component analysis of the transcriptome data. Symbols represent +Fe/-H_2_O_2_ (○), +Fe/+H_2_O_2_ (●), -Fe/-H_2_O_2_ (□) and -Fe/+H_2_O_2_ (■) cultures

**Fig. 2:**
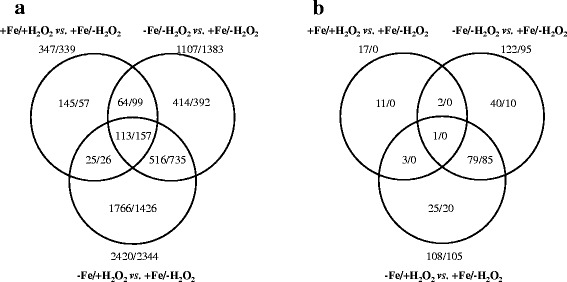
Venn-diagrams of the detected changes in the transcriptome and proteome. Graphs indicating the number of up-regulated/down-regulated (**a**) genes and (**b**) proteins

In order to test how iron deprivation modifies the oxidative stress response of *A. fumigatus*, the transcriptome (Fig. 1, Table 1) and proteome of four different cultures, -Fe/-H_2_O_2_ (iron-deprived), +Fe/+H_2_O_2_) (H_2_O_2_-treated), -Fe/+H_2_O_2_ (iron-deprived-H_2_O_2_-treated), and + Fe/-H_2_O_2_ (iron-replete) cultures were studied in three biological replicates. The -Fe/-H_2_O_2_ and the -Fe/+H_2_O_2_ conditions resulted in significant changes at both the transcriptome and the proteome level in comparison to +Fe/-H_2_O_2_ control cultures (Fig. 2, Tables 2 and 3).

**Table 1:** Basic statistics of the transcriptome data

Sample	Total reads (10^6^)	Mapped reads (10^6^)	Mapped ratio (%)
+Fe/-H_2_O_2_ 1	19.0	18.3	96.0
+Fe/-H_2_O_2_ 2	15.3	14.7	95.5
+Fe/-H_2_O_2_ 3	14.8	14.2	96.0
Sum:	49.2	47.2	95.9
+ Fe/+H_2_O_2_ 1	18.8	17.9	95.5
+ Fe/+H_2_O_2_ 2	17.1	16.3	95.7
+ Fe/+H_2_O_2_ 3	14.6	14.0	95.8
Sum:	50.5	48.3	95.7
-Fe/-H_2_O_2_ 1	15.0	14.4	95.9
-Fe/-H_2_O_2_ 2	16.3	15.6	95.5
-Fe/-H_2_O_2_ 3	17.6	16.8	95.6
Sum:	48.9	46.8	95.7
-Fe/+H_2_O_2_ 1	16.4	15.8	95.8
-Fe/+H_2_O_2_ 2	18.3	17.6	96.0
-Fe/+H_2_O_2_ 3	16.1	15.5	96.1
Sum:	50.9	48.9	96.0
Total sum:	199.5	191.1	95.8

**Table 2:** Results of the gene enrichment analysis

Comparison	Up-	Down-	Significant enriched FunCat terms^a^	
	regulated genes		for up regulated genes	for down regulated genes
-Fe/-H_2_O_2_ vs. +Fe/-H_2_O_2_	1107	1383	secondary metabolism, drug/toxin transport, homeostasis of metal ions, siderophore-iron transport, degradation of aspartate	translation, Fe/S binding, ribosome biogenesis, secondary metabolism, aerobic respiration, detoxification involving cytochrome P450, heme binding, TCA pathway, metabolism of melanins, catalase reaction
+Fe/+H_2_O_2_ vs. +Fe/-H_2_O_2_	347	339	secondary metabolism	secondary metabolism, siderophore-iron transport, extracellular polysaccharide degradation, homeostasis of metal ions
-Fe/+H_2_O_2_ vs. -Fe/-H_2_O_2_	2125	2028	proteasomal degradation, heat shock response, vacuolar/lysosomal transport, oxidative stress response, DNA repair	ribosome biogenesis, translation, allantoin and allantoate transport, secondary metabolism, vitamin/cofactor transport, fatty acid metabolism, virulence and disease factors, degradation of glutamine
-Fe/+H_2_O_2_ vs. +Fe/-H_2_O_2_	2420	2344	vacuolar/lysosomal transport, heat shock response, proteasomal degradation, transcription initiation, vacuole or lysosome, DNA repair	ribosome biogenesis, translation, Fe/S binding, TCA pathway, secondary metabolism, aerobic respiration, detoxification involving cytochrome P450, allantoin and allantoate transport, disease-virulence-defense, heme binding

^a^Selected significant (p < 0.05) shared FunCat terms are presented. The full data sets, which also contain the significant shared GO and KEGG pathway terms are available in Additional file 2

**Table 3:** Results of the protein enrichment analysis

Comparison	Up	Down	Significant enriched FunCat terms^a^	
	regulated proteins		for up-regulated proteins	for down-regulated proteins
-Fe/-H_2_O_2_ vs. +Fe/-H_2_O_2_	122	95	siderophore-iron transport, oxygen and radical detoxification	Fe/S binding, aerobic respiration, translation, ribosome biogenesis, sulfate assimilation, biosynthesis of leucine, unfolded protein response, biosynthesis of homocysteine, TCA pathway
+Fe/+H_2_O_2_ vs. +Fe/-H_2_O_2_	17	0	No significant term was found.	No proteins were related to this gene group.
-Fe/+H_2_O_2_ vs. -Fe/-H_2_O_2_	3	0	heat shock response, oxidative stress response	No proteins were related to this gene group.
-Fe/+H_2_O_2_ vs. +Fe/-H_2_O_2_	108	105	siderophore-iron transport	Fe/S binding, aerobic respiration, translation, ribosome biogenesis, TCA pathway, biosynthesis of glutamate, heme binding, biosynthesis of leucine, biosynthesis of homocysteine

^a^Selected significant (p < 0.05) shared FunCat terms are presented. The full data sets, which also contain the significant shared GO and KEGG pathway terms are available in Additional file 2

**Fig. 3:**
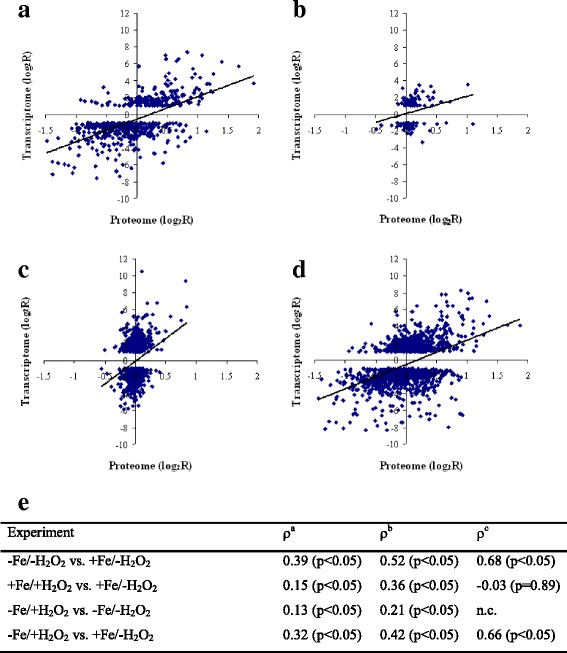
Correlation between proteome and transcriptome data. Data pairs containing the log_2_ ratio (log_2_FC) of the mean FPKM values (in case of the up- or down regulated genes only) and the log_2_ ratio (log_2_FC) of the appropriate mean protein abundance values are presented. A - -Fe/-H_2_O_2_ vs. +Fe/-H_2_O_2_ B - + Fe/+H_2_O_2_ vs. +Fe/-H_2_O_2._ C - -Fe/+H_2_O_2_ vs. -Fe/-H_2_O_2._ D - -Fe/+H_2_O_2_ vs. +Fe/-H_2_O_2._ E - Pairwise Spearman’s rank correlation coefficients calculated from the log_2_FC values (transcriptome vs. proteome). a - all available transcriptome vs. proteome data pairs. b - transcriptome data of up- or down-regulated genes only vs. proteome data. c - proteome data of up- or down-regulated proteins only vs. transcriptome data. n.c. - We found only three up- or down-regulated proteins, therefore a correlation coefficient was not calculated in this case

In contrast, the H_2_O_2_-induced oxidative stress had only a moderate effect on both the transcriptome and the proteome of *A. fumigatus* cultures cultivated under iron-replete conditions (Fig. 2, Tables 2 and 3). It cannot be ruled out that the applied 3 mM concentration of H_2_O_2_ was not sufficient to elicit more significant transcriptomic and proteomics changes. However, higher H_2_O_2_ concentrations were lethal under iron-deprived conditions (data not shown) and, in addition, even a H_2_O_2_ concentration of 2 mM was shown to activate an AfYap1-dependent oxidative stress response in *A. fumigatus* [36]. The recorded changes at the mRNA and protein level showed a relatively good correlation for the comparison of cultures grown under -Fe/-H_2_O_2_ vs. -Fe/+H_2_O_2_ conditions (Fig. 3). Nevertheless, a weaker correlation of the transcriptome and proteome data was found for the +Fe/+H_2_O_2_ vs. +Fe/-H_2_O_2_ and the –Fe/+H_2_O_2_ vs. -Fe/-H_2_O_2_ cultures (Fig. 3).

### Characterization of the stress responsive genes/proteins

To understand the physiological changes under the applied stress conditions, the stress responsive genes/proteins were studied by enrichment analysis using FunCat, GO, and KEGG pathway terms (Additional file 2, Tables 2 and 3). The regulations of the following gene/protein groups, which are potentially important for the oxidative stress or iron starvation response and/or the virulence of *A. fumigatus* [6–10], were also studied in detail: (1) “Antioxidant enzymes”; (2) genes/proteins related to iron metabolism including “Iron transport”, “Fe-S cluster assembly”, “Heme biosynthesis”, “Fe-S cluster binding”, “Heme binding”, “TCA cycle”, “Respiration”, “Squalene-ergosterol pathway”; (3) further genes/proteins related to the virulence including “Cu-transport”, “Zn-transport”, “Drug transmembrane transport” and “Secondary metabolite cluster genes”; as well as (4) “Transcription factors” (Additional files 3 and 4). For selected genes, the transcriptional changes were also confirmed by RT-qPCR (Additional file 5).

### Effects of iron deprivation (-Fe/-H_2_O_2_ vs. +Fe/-H_2_O_2_)

Enrichment analyses of stress responsive genes and proteins under iron-deprived conditions suggested up-regulation of siderophore metabolism, alterations in secondary metabolism and amino acid metabolism, as well as down-regulation of translation, formation of heme and FeS cluster proteins, TCA cycle and mitochondrial respiration (Additional files 2, 3, 4 and 5, Tables 2 and 3).

**Fig. 4:**
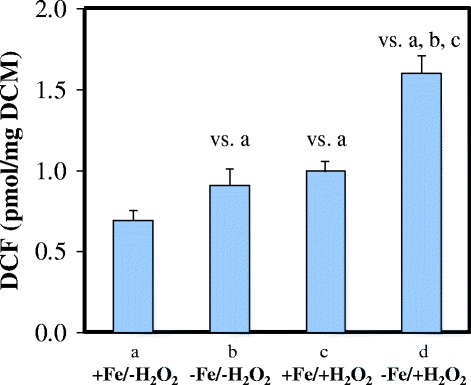
DCF production of the –Fe/+H_2_O_2_ *A. fumigatus* cultures. Redox imbalance caused by stress treatment was quantified with the 2′,7′-dichlorofluorescin diacetate assay. DCF productions were given as produced pmol DCF / mg dry cell mass (DCM). Mean ± S.D. calculated from three independent experiments are presented. The label “vs. a”, “vs. b”, and “vs. c” represent significantly increased DCF production compared to iron-repleted (+Fe), iron-depleted (-Fe), and H_2_O_2_-treated iron-replete (+Fe/+H_2_O_2_) cultures, respectively, according to Student’s t-test (*p* < 0.05)

Iron deprivation caused alterations in the antioxidant defense system of *A. fumigatus*. Down-regulation of the iron-containing enzymes Cat1 and Ccp1 (peroxidase) were detected on the transcriptome and proteome level, while certain iron-free antioxidant enzymes i.e. Trr1 (putative thioredoxin reductase), Afu5g11320 (putative thioredoxin), and Sod1 (CuZn-SOD) were up-regulated (Additional files 3 and 5). Iron deprivation also induced alterations in the redox status of *A. fumigatus* as visualized by the ROS-activated fluorescent probe 2′,7′-dichlorofluorescein diacetate (Fig. 4).

Up-regulation of siderophore metabolism (siderophore biosynthesis and ferri-siderophore uptake) was accompanied by the up-regulation of *amcA* and *hmg2*, which are both involved in supplying precursors for siderophore biosynthesis (Additional file 3). The *amcA* gene encodes a transporter involved in the mitochondrial export of L-ornithine [59], which is the main precursor of siderophore biosynthesis in *A. fumigatus*. The gene *hmg2* encodes the enzyme hydroxymethylglutaryl-CoA reductase, which catalyzes the formation of mevalonate, a precursor of the extracellular siderophores fusarinine C and triacetyl-fusarinine C (TAF-C) [60]. Moreover, synchronous up-regulation of the iron regulator *hapX* [35] and down-regulation of the GATA-factor *sreA* (a repressor of siderophore biosynthesis [61, 62]) were also observed (Additional files 3 and 5). Elevated siderophore production was also confirmed by the CAS assay; 49 ± 6 μM (*n* = 4) of extracellular siderophores (50 h) were detected in iron-starved cultures, while < 3 μM was measured in iron-replete cultures.

It is notable that although several Fe-S cluster- and heme-binding proteins were down-regulated (Additional files 3 and 5) together with a few important genes of Fe-S cluster assembly (e.g. *isa1* [35]) and heme biosynthesis (e.g. *hemA* [35]), no bulk down-regulation of genes belonging to these processes was observed (Additional file 3). Moreover, up-regulation of genes encoding iron-dependent ergosterol biosynthesis enzymes (*erg3A, erg3B, erg25A, erg25B*) (Additional files 3) and corresponding increased levels of the enzyme Erg3A was detected (Additional file 3). In contrast, we could not identify the up-regulation of any other iron-dependent, ergosterol biosynthetic enzymes, including Erg5 (sterol C22 desaturase), Cyp51A and B (lanosterol 14α-demethylases), or their corresponding genes (Additional file 3).

Up-regulation of drug transmembrane transport genes as well as the down-regulation of Zn^2+^ and Cu^2+^ transport genes was also observed (Additional file 3). When one considers the stress-dependent expression of secondary metabolite biosynthetic genes, both up- and down-regulated genes that fall into this category were significantly enriched under iron deprivation (Additional files 4 and 5). Five secondary metabolite biosynthetic gene clusters (fumagillin, siderophore, hexadehydro-astechrome, pseurotin A, and Afu3g02670 clusters) showed significant up-regulation, while ten clusters (DHN-melanin, endocrocin, fumigaclavine C, fumipyrrole, fumiquinazoline, fumitremorgin B, gliotoxin, Afu3g13730, and Afu6g13930 clusters together with the “No PKS or NRPS backbone 6” cluster) showed significant down-regulation (Table 4).

**Table 4:** Regulation of selected secondary metabolism biosynthetic gene clusters

Cluster	Cluster size^a^	Related genes (up-regulated/down-regulated)			
		-Fe/-H_2_O_2_ vs. +Fe/-H_2_O_2_	+Fe/+H_2_O_2_ vs. +Fe/-H_2_O_2_	-Fe/+H_2_O_2_ vs. -Fe/-H_2_O_2_	-Fe/+H_2_O_2_ vs. +Fe/-H_2_O_2_
DHN-melanin cluster	10	1/8^b^	1/0	3/0	2/7^b^
Endocrocin cluster	9	2/4^b^	5^b^/1	1/4	1/5^b^
Fumagillin cluster	15	8^b^/0	11^b^/0	0/15^b^	1/2
Fumigaclavine C (fga) cluster	11	0/5^b^	4^b^/0	0/1	0/6^b^
Fumipyrrole cluster	7	0/7^b^	0/0	2/1	1/7^b^
Fumiquinazoline cluster	5	0/5^b^	1/0	1/0	0/4^b^
Fumitremorgin B (ftm) cluster	9	0/4^b^	0/0	0/7^b^	0/8^b^
Siderophore cluster	18	10^b^/5	0/10^b^	9^b^/3	11^b^/3
Gliotoxin (gli) cluster	12	0/12^b^	2/0	4/1	0/12^b^
Hexadehydro-astechrome cluster	8	7^b^/0	5^b^/0	1/4	3/0
Pseurotin A cluster	4	3^b^/0	4^b^/0	0/4^b^	0/1
Afu1g01010 cluster	4	2/0	1/0	0/4^b^	0/1
Afu3g01410 cluster	9	2/0	2^b^/0	3/4	2/3
Afu3g02570 and Afu3g02530 clusters	15	4/3	0/2	7^b^/0	6/2
Afu3g02670 cluster	7	3^b^/0	0/1	1/2	3/0
Afu3g13730 cluster	9	0/5^b^	0/4^b^	0/1	0/5^b^
Afu5g10120 cluster	10	3/0	1/0	2/0	6^b^/0
Afu6g13930 cluster	9	0/9^b^	0/2^b^	1/5^b^	0/9^b^
Afu7g00170 cluster	7	1/1	4^b^/0	1/0	1/0
No PKS or NRPS backbone 6 cluster	13	1/5^b^	0/2	3/1	3/5
Number of up-regulated clusters		5	7	2	2
Number of down-regulated clusters		10	3	5	9

^a^Number of genes belonging to the cluster. The full data sets are available in Additional file 4

^b^Significantly enriched gene group according to the Fisher’s exact test (p < 0.05)

### Effect of H_2_O_2_ treatment on iron-replete cultures (+Fe/+H_2_O_2_ vs. +Fe/-H_2_O_2_ cultures)

The employed oxidative stress treatment caused significant alterations in the redox homeostasis of iron-replete hyphae (Fig. 4). However, functional analysis of up- and down-regulated genes resulted in few significant shared FunCat, GO, or KEGG pathway terms, which reflected changes in secondary metabolism and also indicated down-regulation of siderophore biosynthesis (Table 2, Additional file 2) and Cu^2+^ transport (Additional file 3). At the level of proteome, only the GO terms “hydrogen peroxide catabolic process” and “removal of superoxide radicals” were significantly enriched. However, these two biological processes incorporated only two proteins: the putative thioredoxin reductase Trr1 and the catalase Cat2 (Additional files 2 and 3).

Similar to iron-deprived conditions, both up- and downregulated genes involved in the biosynthesis of secondary metabolites were significantly enriched (Additional file 4): Seven gene clusters (endocrocin, fumagillin, fumigaclavine C, hexadehydro-astechrome, pseurotin A, Afu3g01410, and the dimethylallyl tryptophan synthase containing Afu7g00170 cluster) showed significant up-regulation, meanwhile only three clusters (siderophore, Afu3g13730, and Afu6g13930) showed concomitant down-regulation (Table 4, Additional files 4 and 5).

### Combined effects of iron-deprivation and H_2_O_2_ treatment on control cultures (-Fe/+H_2_O_2_ vs. +Fe/-H_2_O_2_)

H_2_O_2_-treatment of iron-deprived cultures perturbed significantly the redox homeostasis of hyphae (Fig. 4) and, concomitantly, induced the expression of a group of DNA repair, heat shock and oxidative stress response genes, which coincided with the induction of *atfA*, *yap1,* and *hsf1* genes (Table 2, Additional files 2 and 3) coding for important transcription factors governing oxidative and heat stress responses in *A. fumigatus* [21, 25, 36, 63]. Macroautophagy, ubiquitin-dependent protein degradation (Additional file 2), as well as siderophore metabolism and drug transmembrane transport were also induced, meanwhile the down-regulations of genes involved in translation, ribosome biogenesis, TCA cycle, the squalene-ergosterol biosynthesis pathway, respiration, and Zn^2+^ transport were detected (Table 2, Additional files 2 and 3).

Regarding secondary metabolism, the Afu5g10120 and siderophore clusters showed up-regulation, whereas nine clusters (DHN-melanin, endocrocin, fumigaclavine C, fumipyrrole, fumiquinazoline, fumitremorgin B gliotoxin, Afu3g13730, and Afu6g13930 clusters) were down-regulated (Table 4, Additional files 4 and 5).

Although the down-regulated genes encoding heme binding and Fe-S cluster binding proteins were significantly enriched under combined stress conditions, many other genes of this category (12 and 12 genes, respectively), together with 7 genes from the “Fe-S cluster assembly” group - showed up-regulation (Additional file 3). Importantly, these up-regulations were exclusively detected at the transcript level (Additional file 3). Only the Afu2g14960 gene, which encodes a putative Fe-S binding protein, showed a concomitant up-regulation on the protein level (Additional file 3).

### Effect of H_2_O_2_-induced oxidative stress on iron-deprived cultures (-Fe/+H_2_O_2_ vs. -Fe/-H_2_O_2_)

To better understand the impact of H_2_O_2_ on iron-deprived cultures, we directly compared the transcriptome and the proteome of *A. fumigatus* grown under -Fe/+H_2_O_2_ and -Fe/-H_2_O_2_ conditions (-Fe/+H_2_O_2_ vs. -Fe/-H_2_O_2_). In addition, we addressed the question of whether the effect of H_2_O_2_ differs between iron-replete cultures (+Fe/+H_2_O_2_ vs. +Fe/-H_2_O_2_) and iron-deprived cultures (-Fe/+H_2_O_2_ vs. -Fe/-H_2_O_2_).

The changes detected in the functionally-related gene groups during the comparison of -Fe/+H_2_O_2_ and -Fe/-H_2_O_2_ cultures were generally similar to those found in the -Fe/+H_2_O_2_ vs. +Fe/-H_2_O_2_ comparison (Tables 2, 3 and 4, Additional files 2, 3 and 4). The major differences were as follows: neither iron transport genes nor Zn^2+^ transport genes showed further transcriptional changes in comparison to the iron-deprived cultures (Additional file 3). Surprisingly, the Fe-S cluster assembly genes showed clear up-regulation, meanwhile the drug transmembrane transport genes were down-regulated in comparison to -Fe/+H_2_O_2_ and -Fe/-H_2_O_2_ cultures (Additional files 3 and 5). The genes of Fe-S cluster binding proteins and genes involved in respiration were not down-regulated (Additional file 3). Moreover, many of them showed up-regulation. However, this group of genes was not significantly enriched (Additional file 3). The repression of several other “iron-dependent” genes during iron deprivation was also overruled, at least partially, by oxidative stress induction. Prominent examples are the aconitase-encoding gene *acoA*, the heme biosynthesis gene *hem13* and the catalase genes *cat1* and *cat2* (Additional file 3).

**Fig. 5:**
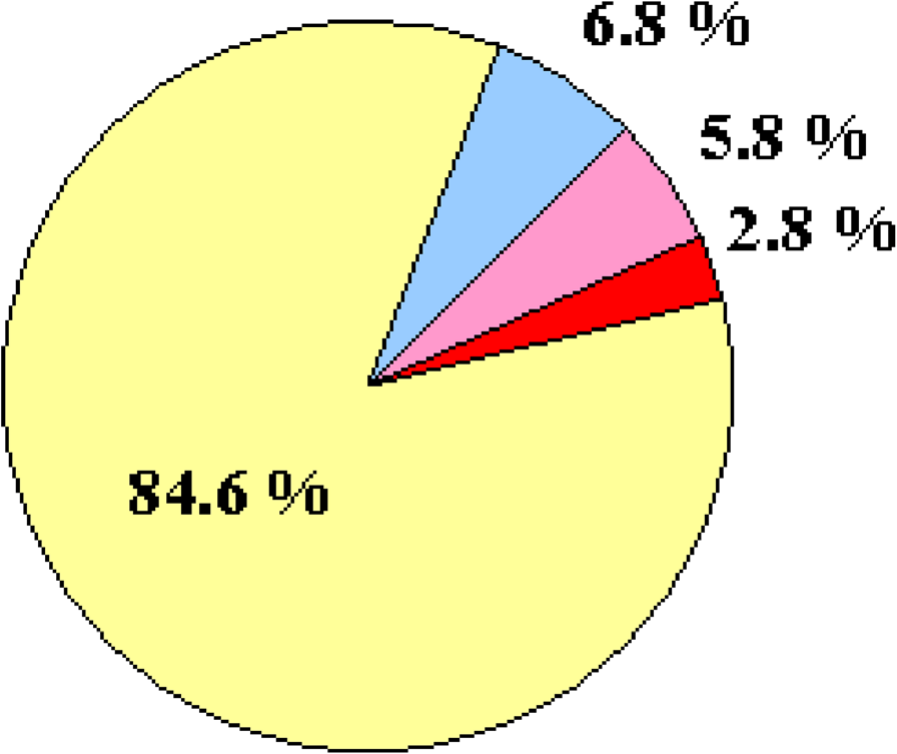
Comparison of oxidative stress response in iron-replete (+Fe/+H_2_O_2_ vs. +Fe/-H_2_O_2_) and iron-depleted cultures (-Fe/+H_2_O_2_ vs. -Fe/-H_2_O_2_). Percentages of stress responsive genes (differentially expressed genes with at least two-fold transcriptional difference) detected only in iron-depleted cultures (yellow), only in iron-replete cultures (blue), in both cultures with unidirectional transcriptional changes (pink), and in both cultures with opposite transcriptional changes (red) are presented

Addition of H_2_O_2_ to iron-deprived cultures caused very significant transcriptional changes different from that found in iron-replete cultures (Tables 1 and 2, Fig. 1). Both the number and function of stress responsive genes were different (Table 2) and only 5.8% of stress responsive genes showed similar (unidirectional) transcriptional changes in both cultures challenged with H_2_O_2_ (+Fe/+H_2_O_2_ vs. +Fe/-H_2_O_2_ and –Fe/+H_2_O_2_ vs. -Fe/-H_2_O_2_) (Fig. 5). The regulation of genes coding for antioxidative enzymes illustrates nicely the dependence of the oxidative stress response on iron availability: in total 12 antioxidative enzyme genes showed down-regulation under iron deprivation. Five of them (*cat2* and *aspf29* encoding a putative bifunctional catalase-peroxidase and a putative thioredoxin, respectively, Afu3g12270 and Afu3g12270 coding for putative glutathione peroxidases, and also the Afu5g15070 peroxiredoxin gene) were up-regulated under combined stresses. In contrast, H_2_O_2_-induced oxidative stress alone did not induce their expression (Additional file 3). In addition, a group of 11 genes was up-regulated under combined stress conditions; however, they were not stress responsive upon exposure to a single stress treatment (Additional file 3).

### Iron deprivation decreases the resistance to oxidative stress

**Fig. 6:**
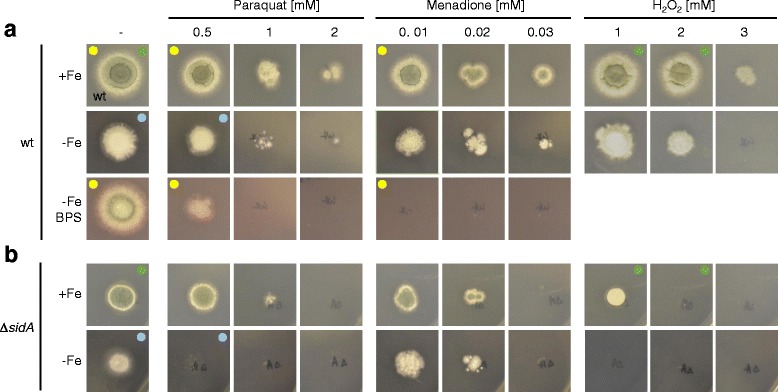
The impact of iron supply on oxidative stress resistance of *A. fumigatus*. *A. fumigatus* wild type and *ΔsidA* strains were point-inoculated on minimal medium plates reflecting different iron supply with and without stressors leading to oxidative stress (H_2_O_2_ and the redox cyclers paraquat and menadione) or BPS, a ferrous iron-specific chelator, which inactivates reductive iron assimilation [30]. Growth was scored after incubation for 48 h at 37 °C. The effect of H_2_O_2_ in the presence of BPS is not shown because H_2_O_2_ interferes with BPS function. The *A. fumigatus ΔsidA* mutant [30] lacks siderophore biosynthesis, which results in decreased iron uptake and decreased resistance to iron starvation. As BPS blocks the growth of *ΔsidA* [30]*,* this mutant was not analyzed in the presence of BPS. Compared to iron sufficiency (+Fe), iron deprivation (-Fe/BPS) increased the oxidative stress accessibility of the wild type strain (marked by yellow dots); compared to the wild-type strain, SidA-deficiency increased oxidative stress accessibility on +Fe medium (marked by green dots) and -Fe medium (marked by blue dots).

To investigate the link between iron supply and oxidative stress at the physiological level, we analyzed the growth response of *A. fumigatus* to oxidative stress in dependence on iron supply. As shown in Fig. 6a, iron deprivation in combination with the ferrous iron-specific chelator BPS, which inhibits reductive iron assimilation, significantly increased the susceptibility of *A. fumigatus* wild type to oxidative stress caused by the redox cyclers paraquat and menadione; i.e. radial growth of the wild-type strain was similar on +Fe and -Fe/BPS media but the growth inhibition mediated by paraquat and menadione was significantly higher in -Fe/BPS compared to +Fe (e.g. 0.5 mM paraquat and 0.01 mM menadione had a negligible effect on +Fe medium but caused drastic growth reduction and growth inhibition, respectively, on -Fe/BPS medium). In agreement, the loss of siderophore-mediated iron uptake (*ΔsidA* mutant) decreased resistance to these oxidative stress-causing reagents (Fig. 6b), e.g. on +Fe medium, 1 mM and 2 mM H_2_O_2_ had minor effects on the wild-type strain but caused significant growth reduction and growth inhibition, respectively, of the *ΔsidA* mutant; similarly, on -Fe medium, 0.5 mM paraquat had a negligible effect on the control strain but blocked growth of the *ΔsidA* mutant. Taken together, these data support our view – drawn from the transcriptome and proteome data – that iron deprivation aggravates oxidative stress.

## Discussion

Iron is an essential transition metal nutrient for all living organisms including *A. fumigatus*. The accessibility of iron plays a crucial role during *A. fumigatus* infections [16, 59] and, not surprisingly, several elements of its iron metabolism have a determinative impact on the virulence of this fungus [30, 32–35]. However, during fungus-host interactions, *A. fumigatus* is exposed to combinatorial stress including confrontation with ROS. In this study, we demonstrated that the response to combinations of stresses (iron depletion plus oxidative stress) differs considerably to the response to a single stress (iron depletion or oxidative stress alone).

### Adaptation to iron-deprivation

The secretion of soluble iron-chelating siderophores under iron depletion is a well-studied mechanism in fungi. The up-regulation of siderophore metabolism under iron deprivation was underlined by both transcriptome and proteome data (Tables 2 and 3, Additional file 3). This is in good accordance with the results of previous studies [35, 59, 62, 64]. Interestingly, the up-regulation of RIA elements (FetC, FreB, FtrA; [30, 61]) was not as obvious as it was in the case of siderophore metabolic enzymes, transporters, and regulators (Additional files 3 and 5).

The up-regulation of iron uptake was accompanied by several processes in order to economize and prioritize iron utilization including:

i) Repression of several elements of the mitochondrial electron transport chain as well as down-regulation of elements of the TCA cycle, like the iron-containing aconitase and succinate dehydrogenase, were observed (Additional file 3), which is in line with previous observations made by Schrettl et al. [35]. Restriction of these mitochondrial pathways represents an important response to iron starvation because mitochondria are the major iron-containing organelle found in fungal cells [65].
ii) Ribosome biogenesis and translation were also repressed in good accordance with earlier results of Schrettl et al. [35], and it was accompanied with down-regulation of the iron-sulfur protein Rli1 (Tables 2 and 3; Additional files 2 and 3). In *Saccharomyces cerevisiae*, the biogenesis of cytosolic ribosomes requires Rli1 and, as a consequence, this process depends on both iron availability and Fe-S cluster assembly [66]. Our data suggest that the orthologue of baker’s yeast Rli1 may link ribosome biogenesis and mitochondrial Fe-S cluster assembly in *A. fumigatus* as well.
iii) Altogether 21 Fe-S cluster and 22 heme-binding proteins were down-regulated (Additional files 3 and 5).

The down-regulation of iron-dependent antioxidant enzymes (e.g. Cat1, Ccp1) was likely counterbalanced by the up-regulation of the iron-free proteins Trr1 (putative thioredoxin reductase), Afu5g11320 (putative thioredoxin), and Sod1 (CuZn-SOD) (Additional file 3). Up-regulation of Sod1 is in good accordance with the earlier results of Oberegger et al. [67]. Importantly, Sod1 is likely to protect Fe-S cluster proteins from the deleterious effects of superoxide [67, 68]. As expected, iron deprivation perturbed the redox balance of cells (Fig. 4) because both catalase and peroxidase activities were reduced, while respiration was impaired.

Interestingly, certain iron-dependent processes, e.g. sterol biosynthesis (the squalene-ergosterol pathway), were even up-regulated (Additional file 3). Earlier studies demonstrated that iron starvation decreased the sterol content of cells [64], which was explained by two major reasons. First, mevalonate is an intermediate of both TAF-C and sterol biosynthesis [59, 60] and, as a consequence, intensified TAF-C production decreases the metabolic flux towards sterols. Second, iron is needed for sterol biosynthesis and, therefore, iron starvation decreases the abundance/activity of certain ergosterol biosynthesis enzymes [60]. To prevent dangerous decreases in the sterol content, fungal cells need to maintain a balance between TAF-C production and sterol biosynthesis, which may be achieved by the induction of the squalene-ergosterol pathway genes. The up-regulation of the aforementioned ergosterol biosynthesis genes together with the observation that no bulk down regulation of Fe-S cluster assembly and heme biosynthesis genes was observed (Additional file 3) suggests that iron starvation does not cause down-regulation of all iron-dependent enzymes/pathways but rather leads to a reprogramming to ensure survival under limited iron supply by prioritization of iron use.

### Effect of oxidative stress on iron-deprived cultures

The applied H_2_O_2_ treatments had only minor effect on iron-supplemented cultures (Tables 2 and 3, Additional files 2, 3 and 4). However, the very same exposures to H_2_O_2_-elicited oxidative stress were clearly more harmful in iron-deprived cultures: Under the combination of H_2_O_2_ and iron-deprivation stresses, i) many more genes were induced or repressed than in iron-replete cultures (Table 2), ii) the bulk up-regulation of DNA repair, heat shock, and oxidative stress response genes was observed, which coincided with up-regulation of genes involved in macroautophagy and ubiquitin-dependent protein degradation (Additional file 2, Table 2), iii) DCF production as a marker of redox imbalance in H_2_O_2_-treated and iron-depleted cultures was significantly higher than that observed in H_2_O_2_-treated iron-replete cultures (Fig. 4), and iv) physiological experiments also demonstrated that iron deprivation can reduce oxidative stress tolerance (Fig. 6). In this regard, it is particularly interesting that, as an example, carbon starvation stress did not decrease but even increased oxidative stress tolerance in yeast [69]. Our data shed light on the severity of combined oxidative and iron-deprivation stress in *A. fumigatus*, which highly supports the view that the combination of withholding iron from pathogens and attacking them with ROS is a highly efficient and, therefore, quite widespread strategy to prevent infections in mammalian hosts [16, 42, 43].

**Fig. 7:**
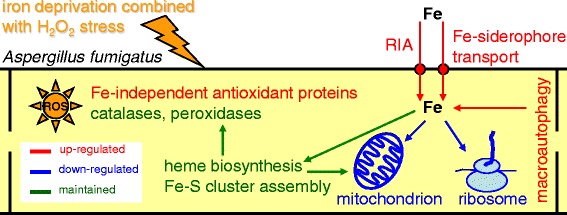
Simplified model of adaptation mechanism to H_2_O_2_ stress under iron starvation in *A. fumigatus* based on the obtained proteomics and transcriptomics data

Under –Fe/+H_2_O_2_ stress conditions, *A. fumigatus* set into operation the following stress response processes to avoid or at least mitigate the deleterious effects of such combinatorial stresses (Fig. 7):

i) Several elements of the glutathione-glutaredoxin-thioredoxin systems were induced and SODs were up-regulated (“iron-independent antioxidant enzymes”), meanwhile DNA repair, heat shock response, macroautophagy, and ubiquitin-dependent protein degradation genes were activated to protect the cells against ROS and to repair the damage caused by ROS under iron-depleted culture conditions (Table 2, Additional file 3).
ii) No further increases in the transcription of siderophore metabolic genes were detected in comparison to –Fe vs. +Fe treatments (Additional file 3). However, iron-deprivation and oxidative stress together clearly up-regulated the expression of RIA genes (Additional files 3 and 5).
iii) The production of iron-containing proteins followed interesting regulatory patterns. While iron deprivation down-regulated a significant group of “Fe-S cluster binding” and “Heme-binding” protein genes (21 and 22 genes, respectively), H_2_O_2_ treatment induced several genes in the same group (11 and 12 genes, respectively) under iron deprivation conditions (Additional file 3). Importantly, these changes were observed at the level of the transcriptome but not the proteome in almost all cases (Additional file 3). This may imply that the up-regulation of these genes is unable to increase the protein levels in the absence of sufficient amount of iron, at least during short-term response. However, preventing any further decrease in their quantities is also a favorable outcome of the up-regulation. Since ROS can inactivate Fe-S clusters [70], the up-regulation of the related genes may also indicate the effort made by the fungus to repair this defect. The successful maintenance of iron-containing protein pools can be an important or even one of the key elements of a successful long-term adaptation to iron starvation. Because the availability of iron is limited under these circumstances fungal cells may also use intracellular iron released by macroautophagy or ubiquitin-dependent protein degradation in addition to iron normally taken up via the siderophore-mediated or RIA systems. In this respect, iron starvation resembles carbon starvation, under which cells release several carbohydrate-active enzymes (CAZymes) to obtain nutrients from their environment and also activate macroautophagy as well as cell wall degrading enzymes to re-utilize the cells’ own building materials [71, 72]. Importantly, using a *Δatg1* mutant, which was impaired in autophagy, Richie et al. [73] clearly demonstrated that autophagy contributed to the recycling of metal ions including iron to maintain vegetative growth under nutrient limitations.
iv) No clear repression of the squalene-ergosterol pathway was observed under combined stresses. Although down-regulated genes (10 genes) were significantly enriched under these conditions in comparison to iron-replete cultures, other genes (8 genes) showed up-regulation in this pathway (Additional files 3). These observations suggest the essentiality of low concentrations of sterols even under –Fe/+H_2_O_2_ stress.
v) Ribosome biogenesis and translation genes were repressed further when compared to –Fe/-H_2_O_2_ vs. +Fe/-H_2_O_2_ treatment (Table 2). We assume that the reduced growth observed under combined stresses led to a decrease in the iron requirement of the cells and shifted the cells’ energy production and assimilatory pathways towards the protection against oxidative stress.

The stress response observed in cultures exposed to iron-starvation/H_2_O_2_ stress (-Fe/+H_2_O_2_ vs. +Fe/-H_2_O_2_) was not a simple combination of iron deprivation (-Fe/-H_2_O_2_ vs. +Fe/-H_2_O_2_) and oxidative (+Fe/+H_2_O_2_ vs. +Fe/-H_2_O_2_) stress responses. Instead, the majority of the observed changes was unpredictable and was only characteristic for the combined stresses (Fig. 5). Owens et al. [74] studied the effect of H_2_O_2_ and gliotoxin treatments on the proteome of *A. fumigatus*. They could also identify several significantly differentially abundant proteins characteristic for the combined (H_2_O_2_/gliotoxin) treatments [74]. We can therefore assume that *A. fumigatus* successfully evolved an appropriate strategy to cope with combined iron starvation--oxidative stress. These data demonstrate that answers to single stress conditions do not necessarily reflect the behavior and capabilities in a complex habitat like the human body with versatile and even rapidly changing stress conditions. Therefore, any studies elucidating how a pathogenic microbe responds to combined stresses in the host organism in vivo are particularly fruitful [8]. Similar to the transcriptomics and proteomics data presented here, several studies in *Candida albicans* underlined that the adaptive responses to combinatorial stresses is not equivalent to the sum of the responses to the corresponding individual stresses [75]. In agreement with the gene expression data, growth studies confirmed that in *A. fumigatus* iron starvation aggravates oxidative stress susceptibility. Similar to *A. fumigatus*, decreased iron supply due to siderophore-deficiency decreases oxidative stress resistance in *Cochliobolus heterostrophus* [76].

### Changes in some medically important attributes of *A. fumigatus*

Stress responses alter significantly the physiology of fungal cells, which may also include alterations in the virulence and/or antifungal drug susceptibility of the fungus. Both iron deprivation (–Fe/-H_2_O_2_) and the combined –Fe/+H_2_O_2_ stress affected the transcription of several genes with known or putative functions in the transmembrane transport of drugs (Additional file 3). The majority of the studied drug transmembrane transport genes (17/25 genes) was up-regulated at least under one stress condition; moreover, the up-regulation of the ABC transporter AbcB protein was also observed, when the proteomes of iron-starved or combined stress-exposed cultures were analyzed (Additional file 3). Importantly, AbcB (Cdr1B) found to be involved in *cyp51a*-independent azole resistance in *A. fumigatus* [77]. The applied severe stress treatments may induce an adaptive prediction of possible environmental changes by *A. fumigatus*, similar to that presented and discussed before for *Escherichia coli* and *S. cerevisiae* [78]. The clinical significance of these changes may initiate further investigations, and our results support the relevance of antifungal strategies based on the inhibition of efflux systems operating in fungal cells [79, 80].

Iron starvation negatively affected the transcription of several genes encoding zinc and copper transporters as well (Additional file 3). These changes could simply be triggered by the reduced growth of *A. fumigatus* observed under iron deprivation or there might be an interplay between and co-regulation of the up-take and utilization of various transition metal ions in this fungus. The interplay between iron and zinc homeostasis has been demonstrated to exist in *A. fumigatus* by Yasmin et al. [81]. Supporting our data presented in Additional file 3, these authors demonstrated that iron starvation down-regulated *zrfB* plasma membrane Zn^2+^ transporter and up-regulated *zrcA* vacuolar Zn^2+^ transporter genes. Interestingly, the Zn^2+^ contents of iron-starved mycelia even exceeded those found in control cultures, which was attributed to the likely action of unspecific metal transporters [82]. In addition, overexpression of *zrfB* resulted in higher zinc toxicity under iron-limited than iron-fed conditions [81]. Because the up-take and homeostasis of zinc can be promising targets in the development of new generations of antifungal drugs, a deeper understanding of the elements and regulation of zinc metabolism especially under iron-limited conditions will be an unavoidable and crucially important task in any forthcoming future studies planned to be carried out in this field [17, 81, 82].

Not surprisingly, the tested stress conditions also significantly affected the secondary metabolism (Table 4, Additional file 4). The regulation of the secondary metabolism of fungi by various types of oxidative stress is a well-studied phenomenon [26, 83, 84]. Furthermore, the iron-dependent regulation of certain secondary metabolite gene clusters has also been described in *A. fumigatus* [85]. Nevertheless, the global regulation of the transcription of secondary metabolite gene clusters was not unambiguous in *A. nidulans* cultures subjected to various short-term oxidative stress treatments [86]. Surprisingly, only a group of the secondary metabolite biosynthetic genes responded to stress at all, and both induced and repressed clusters were detected [86]. In this study, iron starvation significantly reduced the transcriptional activity of the gliotoxin cluster, while both iron starvation and H_2_O_2_ treatment, up-regulated the gene clusters encoding biosynthesis of hexadehydro-astechrome, fumagillin, and pseurotin A (Table 4). Both gliotoxin and fumagillin have an immune response modulator activity and, hence, influence the virulence of *A. fumigatus* [14, 87]. Meanwhile, the overproduction of hexadehydro-astechrome is also accompanied with an enhanced virulence [88]. It is important to note that harsh environmental stress conditions seem to generally down-regulate the majority of secondary metabolite gene clusters [86] and, in accordance with our previous findings, the combined iron starvation--oxidative stress did not induce the aforementioned secondary metabolite gene clusters in *A. fumigatus* (Table 4).

## Conclusions

To confront invading pathogen microbes with combined iron starvation and oxidative stress seems to be a widespread and highly efficient strategy of the host organism to prevent the progression of infections. Nevertheless, *A. fumigatus* has the capability to reorganize its stress response system dynamically and effectively to survive complex stresses present in its habitats. In this study, we demonstrated that the adaptation of *A. fumigatus* to iron starvation combined with H_2_O_2_-elicited oxidative stress followed a unique pattern at the level of the transcriptome and proteome, which was basically different from those we observed in cultures solely exposed to either iron-deprivation or oxidative stress. However, our results also indicate that even successful adaptations to severe combined stresses can be fragile. Importantly, perturbations of fungal iron metabolism e.g. via exposing *A. fumigatus* to heme biosynthesis [89] or Fe-S cluster assembly [90, 91] inhibitors may provide us with suitable tools in future antifungal drug research to combat *A. fumigatus* infections. Although both pathways are highly conserved across taxa, some differences between human and fungi exist [92, 93].

## Additional files

Additional file 1: Primer pairs used in the study. (PDF 12 kb)

Additional file 2: Gene/protein enrichment analysis. Significant shared GO, FunCat or KEGG pathway terms were determined with AspGD Gene Ontology Term Finder (http://www.aspergillusgenome.org/cgi-bin/GO/goTermFinder) or FungiFun2 (https://elbe.hki-jena.de/fungifun/fungifun.php). Terms highlighted with yellow are presented in Tables 2 and 3. (XLS 790 kb)

Additional file 3: Regulation of certain gene/protein groups. Up- and down-regulated genes and proteins were defined in the Materials and methods section and were marked with red and blue colors, respectively. Figures represent log_2_FC values, whereby FC is short for “fold-change”. The FC ratios were calculated for the transcriptome data based on FPKM values and for the proteome data based on iTRAQ reporter ion intensities. Results of gene/protein enrichment analysis (Fisher’s exact test) are also enclosed. (XLS 210 kb)

Additional file 4: Regulation of secondary metabolite cluster genes and proteins. Up- and down-regulated genes and proteins were defined in the Materials and methods section and were marked with red and blue colors, respectively. Figures represent log_2_FC values, whereby FC is short for “fold-change”. The FC ratios were calculated for the transcriptome data based on FPKM values and for the proteome data based on iTRAQ reporter ion intensities. Results of gene/protein enrichment analysis (Fisher’s exact test) are also enclosed. (XLS 116 kb)

Additional file 5 Results of RT-qPCR measurements. Relative transcription levels were quantified with ΔΔCP = ΔCPtreated – ΔCPcontrol. ΔCPtreated = CPreference gene - CPtested gene measured from treated cultures. ΔCPcontrol = CPreference gene - CPtested gene measured from control cultures or from the iron depleted cultures. CP values stand for the qRT-PCR cycle numbers of crossing points. The *fks1* gene was used as reference gene. qRT-PCR data are presented as the mean and S.D. data calculated from three measurements. Significantly higher or lower than 0 ΔΔCP values (up- or down-regulated gene) are marked with red and blue colors, respectively (Student’s t-test, *p* < 0.05, *n* = 3). (XLS 42 kb)

## Abbreviations

BPS: Bathophenanthroline disulfonate
CAS: Chrome azurol S
DATS: Dimethylallyl tryptophan synthase
DCF: 2′,7′-dichlorofluorescein
DCM: Dry cell mass
FPKM: Fragments per kilobase per million mapped fragments
LC-MS/MS: Liquid chromatography coupled to tandem mass spectrometry
NRPS: Non-ribosomal peptide synthases
PKS: Polyketide synthase
RIA: Reductive iron assimilation
ROS: Reactive oxygen species
RT-qPCR: Reverse-transcription quantitative real-time polymerase chain reaction
SOD: Superoxide dismutase
TAF-C: triacetylfusarinine C
TCA cycle: Tricarboxylic-acid cycle
TF: Transcription factors
TS: Terpene synthase
