# Supplementary material for: Additional oxidative stress reroutes the global response of Aspergillus fumigatus to iron depletion
Source: BMC Genomics. 2018 May 10;19:357. doi: 10.1186/s12864-018-4730-x (PMC5946477; doi:10.1186/s12864-018-4730-x)
Supplement: Supplementary file 1 — Primer pairs used in the study. (PDF 12 kb) [file 12864_2018_4730_MOESM1_ESM.pdf]

**Additional file 1** Primer pairs used in the study

Afu1g06390:

5´-AAGTCCGAGCGTGAGCGTG-3´ and 5´-GGTGAAAGCGAGCAGAGCG-3´

Afu1g06810:

5´-GCTATCGGTGTCGGTGGTG-3´ and 5´-ACTGGGGGAGAAGGGGAAG-3´

Afu1g15960:

5´-GTGTTTATGCTCTCGGTGATGTC-3´ and 5´-CGGGTTCTTCTTCTTCTCTTCTG-3´

Afu1g16130:

5´-TGCGGGAATGCGAGATGAG-3´ and 5´-AGTGATGTGCGAAGGGAGC-3´

Afu1g17270:

5´-CAGTCAGATGCGGGTAGTTG-3´ and 5´-AAGGAAAAGGAGGTAGCGATG-3´

Afu2g07680:

5´-TCTTCTGTGTGTTGGGTTTGG-3´ and 5´-TTCTGGTGGAGGTAGTTGAG-3´

Afu2g17600:

5´-CTCAACGACACCAACACCAAC-3´ and 5´-TGCTTCAACATCAACAGGACC-3´

Afu3g02270:

5´-TCACTGCGGCTTCTTTCC-3´ and 5´-ATCTCGTTGTCACCTCTGGG-3´

Afu3g03420:

5´-CTTTTATCGTGGCTCATTGCG-3´ and 5´-AACCCTTCATCTTTTGCTTCGTC-3´

Afu3g08160:

5´-GGTCGTTGTTGCCATCGGTG-3´ and 5´-CATCTTCAGGTTGTCGGTGCG-3´

Afu3g12270:

5´-AGTCGTCCTCGTCGTCAAC-3´ and 5´-TTCAGCCCCATAAGTCCAG-3´

Afu3g12920:

5´-GTAACGGGACTCAATGGACAC-3´ and 5´-CAAAAGCGATGGAACAGATG-3´

Afu3g14240:

5´-GGAACACACCACGCTCAATG-3´ and 5´-CAATGCCAAAACGAATGCTG-3´

Afu4g06770:

5´-CTCCTGATGTCGGTCTCGG-3´ and 5´-TAATAGCGTCCTCGGCAAGC-3´

Afu4g09110:

5´-GGCACTGGAGGAAGCAAC-3´ and 5´-GCGGTAGAAGATGTCACGG-3´

Afu5g03790:

5´-TCATCAGCATCAGCAATCCC-3´ and 5´-TCCACCTCAACAATCTTCATCG-3´

Afu5g03800:

5´-TTGACTGGCGGCACATTC-3´ and 5´-TGGAGGGAGGCTTGGTTTC-3´

Afu5g03920:

5´-ATTCCACCACGACCAAAACC-3´ and 5´-CACCGATACCTCCTTCTCCAAC-3´

Afu5g10370:

5´-CGCTACTGGTGTCGGTCG-3´ and 5´-GGAAGGTCAAGGTGGGGTC-3´

Afu5g11260:

5´-GAGACCACATCATCCACCG-3´ and 5´-TTCAAGTTCAGACACGCCAG-3´

Afu5g11760:

5´-GGAGACAGCGGGTGATAAG-3´ and 5´-GAGGGAGGGAGTTGAGTTG-3´

Afu6g03480:

5´-ATCAGCCACTTCTTCCTCAGC-3´ and 5´-ACCTTGTTAGCCACCACCG-3´

Afu6g03890:

5´-CTTGTGCCCCTTCGTGTG-3´ and 5´-GCCAGTAGTTGACCGTGCC-3´

Afu6g04360:

5´-ATCGGCAACAAGCACAACAATGG-3´ and 5´-TCCTCATCTCCTCAAAATCCTCC-3´

Afu6g09660:

5´-GAGGGCACCGATGATGAAG-3´ and 5´-AGGCAGACCAGAATGAGCAAG-3´

Afu6g12400:

5´-GTCAGGTCCCCGTTTCCAAG-3´ and 5´-GGCATCATCCAAGTCCAGGTG-3´

Afu8g00370:

5´-GATACTCTTTCCCGCTGCTG-3´ and 5´-TGACGATGATGACGATGCTAC-3´

Afu8g00490:

5´-GACGCCCTGCCTATCAAG-3´ and 5´-GAAAACCCAACGCTCTGC-3´

Afu8g00540:

5´-GGGCGTGTGTGTCTTCTTC-3´ and 5´-CAATGGCGTATGCTTCTCTG-3´
